# Long-term survival after extended resection combined with pericardiectomy for locally advanced intrahepatic cholangiocarcinoma: a case report

**DOI:** 10.1093/jscr/rjaf454

**Published:** 2025-06-27

**Authors:** Kit-Fai Lee, Innes Y P Wan, Charing C N Chong, Hon-Ting Lok, Eugene Y J Lo, Kenneth S H Chok

**Affiliations:** Department of Surgery, Prince of Wales Hospital, Hong Kong, China; Department of Surgery, Prince of Wales Hospital, Hong Kong, China; Department of Surgery, Prince of Wales Hospital, Hong Kong, China; Department of Surgery, Prince of Wales Hospital, Hong Kong, China; Department of Surgery, Prince of Wales Hospital, Hong Kong, China; Department of Surgery, Prince of Wales Hospital, Hong Kong, China

**Keywords:** intrahepatic cholangiocarcinoma, pericardiectomy, diaphragm resection

## Abstract

A 74-year-old man presented with upper gastrointestinal bleeding. Investigation revealed a locally advanced intrahepatic cholangiocarcinoma (ICC) arose from left liver invading duodenum and diaphragm. Left trisectionectomy combined with wedge duodenal resection, left diaphragmatic and pericardial resection was performed. The diaphragmatic/pericardial defect was closed with Gore-Tex mesh. The patient developed post-operative intra-abdominal collection which resolved with percutaneous drainage. Pathology confirmed a 7 cm ICC, there was evidence of rupture with abscess formation, and adhesion but no direct invasion to duodenum and diaphragm. The resection margin was clear. The patient remained well for over 2 years after surgery when he was noted to have a new 2.3 cm left lower lobe lung lesion. Biopsy of lesion showed mucinous adenocarcinoma, which could be lung primary or metastatic ICC. The lung tumor was successfully treated with stereotactic body radiotherapy. He remained well afterwards and survived for ˃5 years without further recurrence since initial operation.

## Introduction

Intrahepatic cholangiocarcinoma (ICC) is a primary liver cancer which accounts for 15%–20% of primary liver malignancies, it is just second in incidence to hepatocellular carcinoma (HCC) [1]. Surgical resection remains the mainstay of treatment for ICC; however, only 20%–30% of patients are candidates for surgery [2]. Occasionally ICC invades outside liver to surrounding organs. Concomitant en bloc resection is necessary to achieve oncological clearance if adjacent organs are involved [3, 4]. Here we report the long-term result of a case of concomitant pericardiectomy, wedge duodenal resection together with left trisectionectomy for a locally advanced ICC.

## Case report

**Figure 1:**
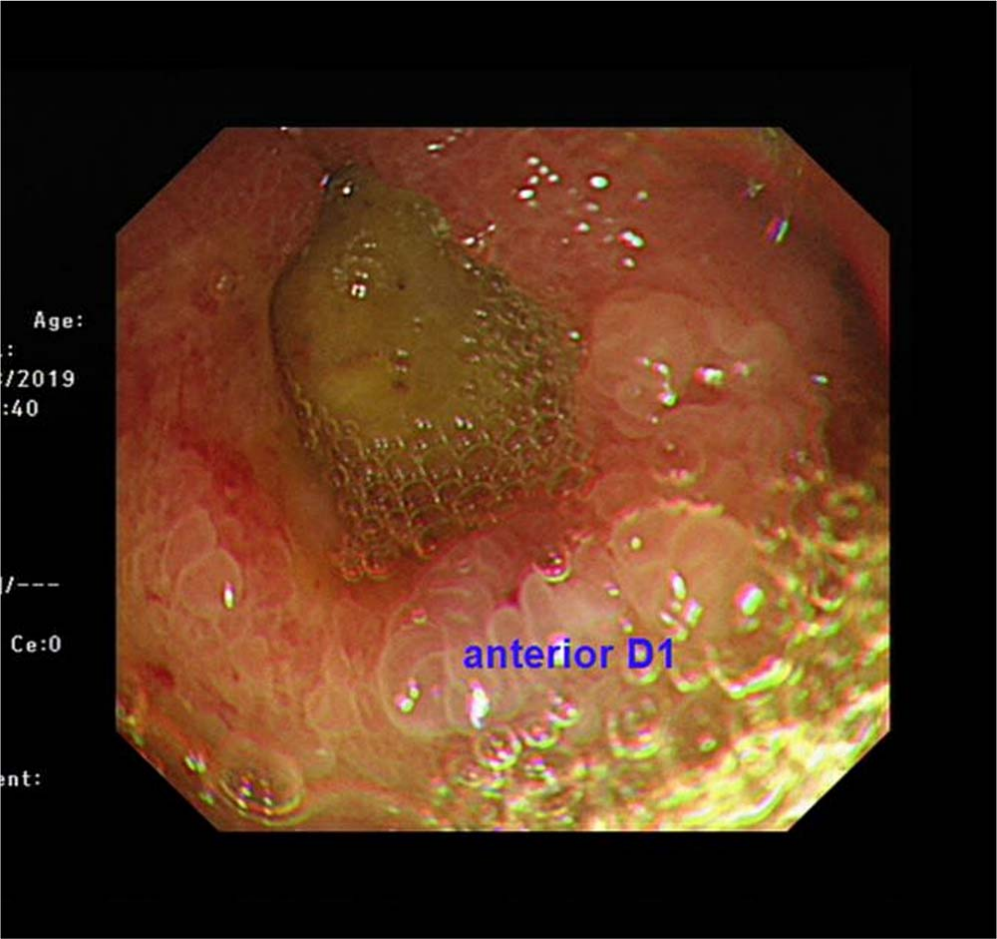
Endoscopic view showing a chronic duodenal ulcer.

**Figure 2:**
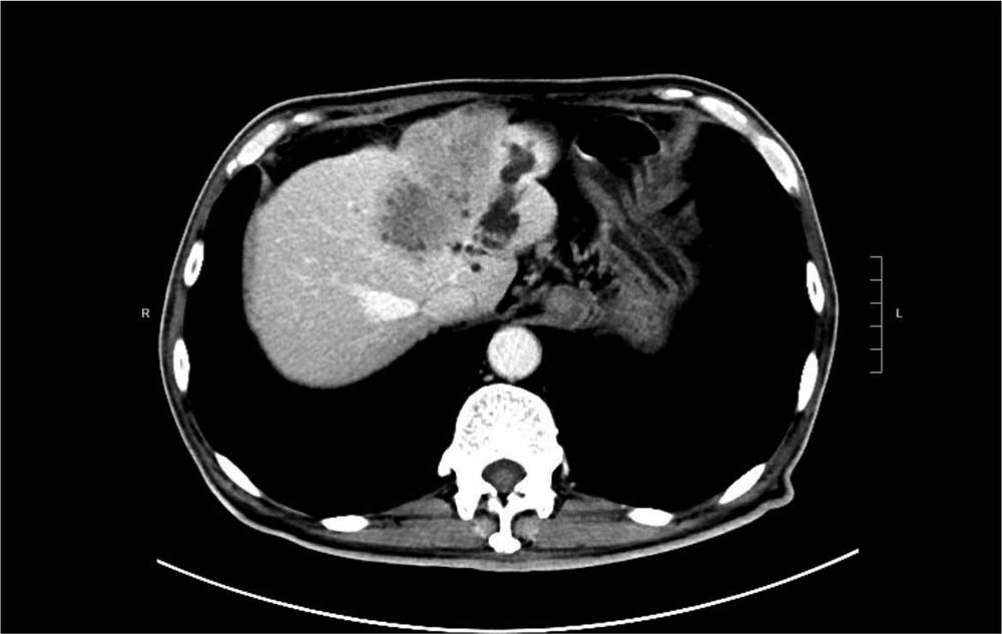
CT showing showing an exophytic hypoenhancing mass at left liver with dilated left intrahepatic duct.

A 74-year-old man who had history of hypertension and diabetes mellitus presented with upper gastrointestinal bleeding. Upper endoscopy revealed deformed pylorus and a 1 cm deep ulcer at anterior wall of first part of duodenum (Fig. 1). Computed tomography (CT) revealed a 4.8 cm exophytic hypoenhancing mass at segment 4a of liver with dilated left intrahepatic duct (Fig. 2). There was aerobilia suggestive of choledochoduodenal fistula. There was also suspicion of diaphragm invasion. Serum carcinoembryonic antigen (CEA) was elevated to 83ug/L while alpha-fetoprotein (AFP) was normal. Both the hepatitis B surface antigen and anti-hepatitis C antibody were negative. Positron emission tomography (PET) confirmed a hypermetabolic liver tumor but no distant metastasis. The clinical diagnosis was ICC with suspected duodenal and diaphragmatic invasion. The patient was offered radical resection for tumor.

**Figure 3:**
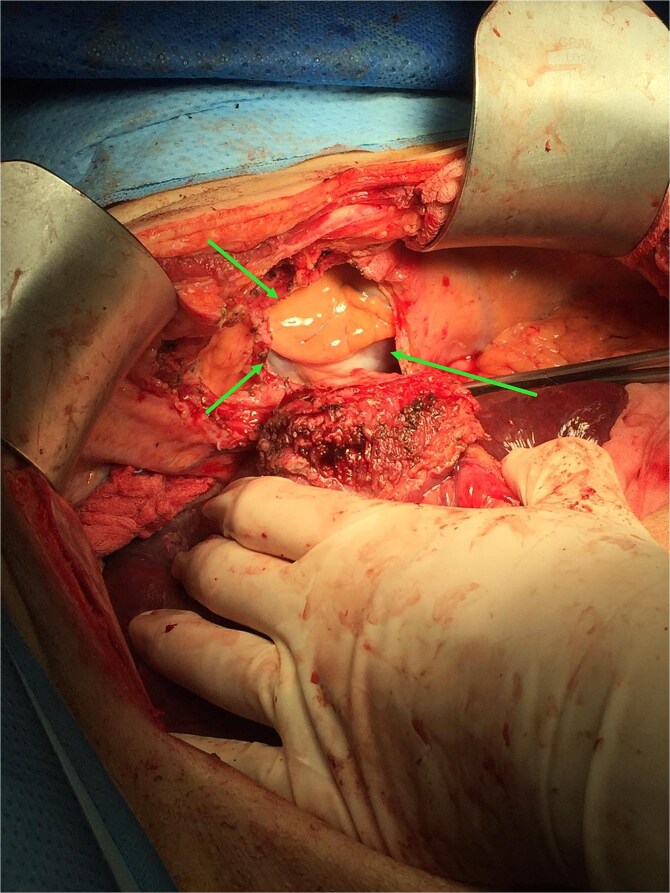
Operative photo showing the pericardium was removed en bloc with liver tumor, the heart was exposed (arrows).

**Figure 4:**
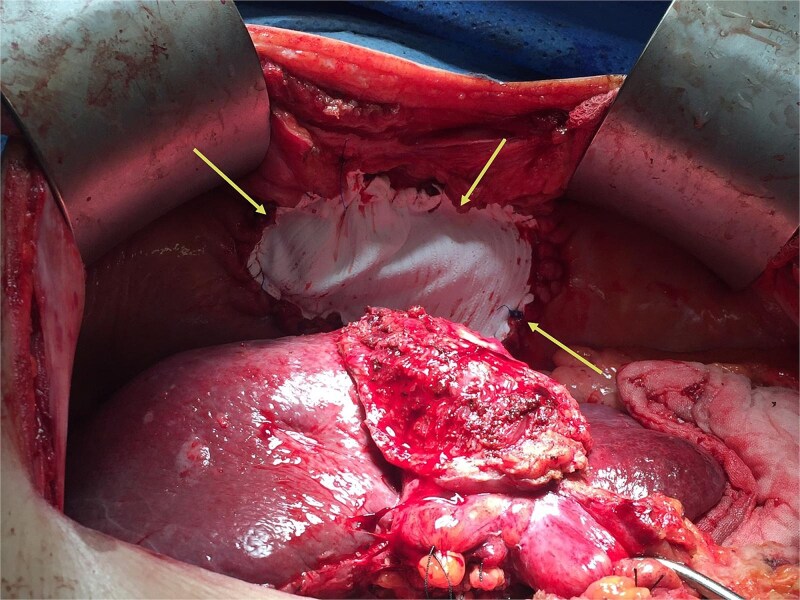
The diaphragm and pericardium was closed with Gore-Tex mesh (arrows).

**Figure 5:**
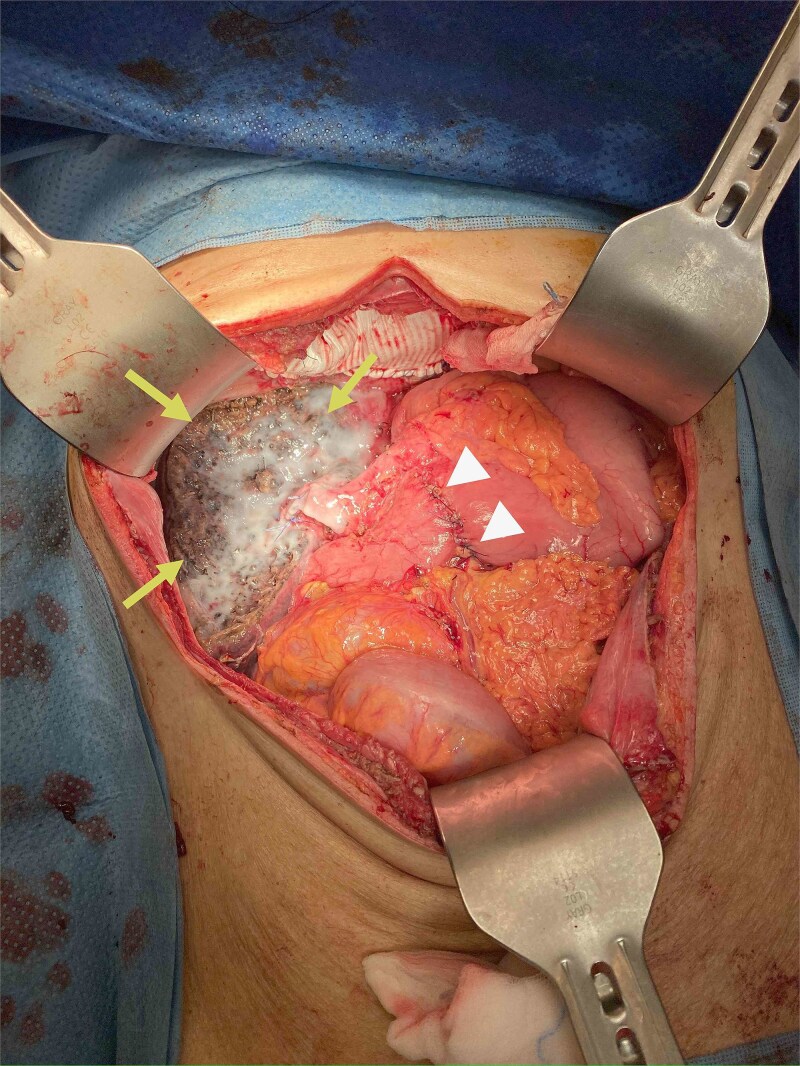
Specimen removed, the transected liver surface was covered with applied tissue glue (arrows) and the duodenal repair site was seen (arrow heads).

**Figure 6:**
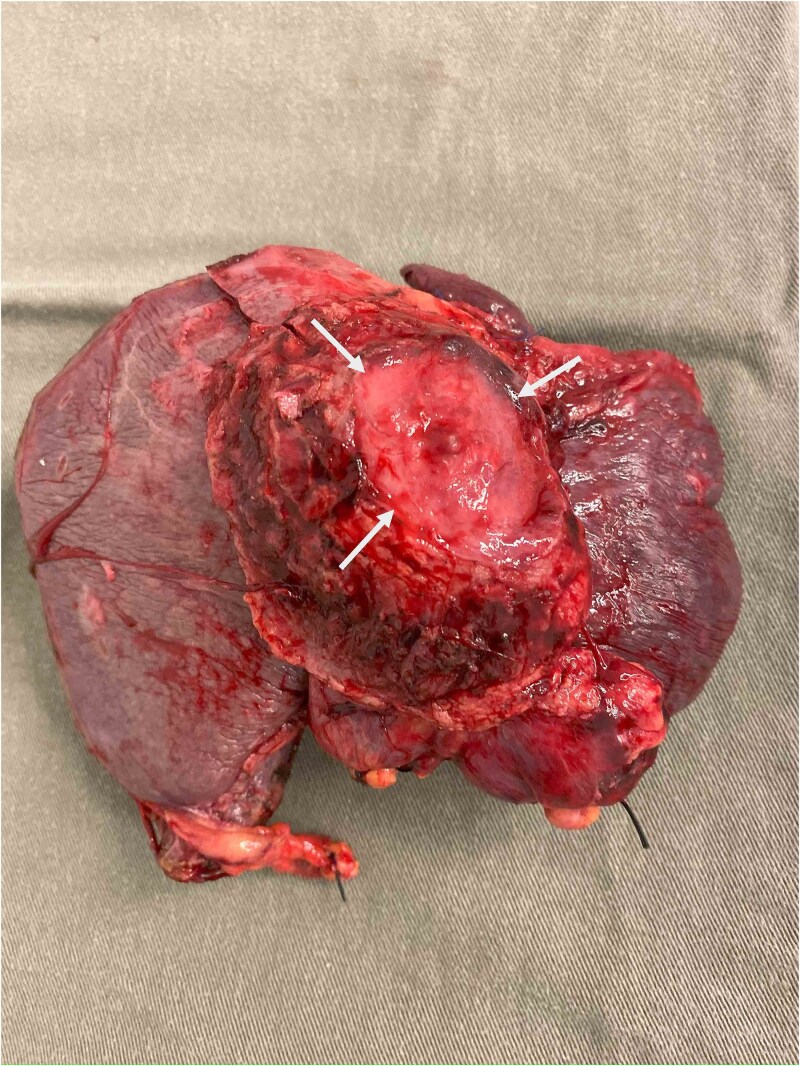
Specimen showing removed liver with attached pericardium (arrows).

During operation, an exophytic 7 cm segment 4 tumor was found invading superiorly to left hemidiaphragm and pericardium, inferiorly invading to first part of duodenum. A cuff of duodenal wall was removed with tumor and the duodenal defect was closed primarily with 3/0 PDS. Part of the left hemidiaphragm together with pericardium was resected, the heart and left lung were exposed (Fig. 3). The diaphragmatic/pericardial defect was closed with Gore-Tex mesh with single layer continuous 3/0 Prolene (Fig. 4). Air was expelled through an under-water seal catheter placed in the pleural cavity before suture was tightened and tied. Left trisectionectomy was then completed (Fig. 5). The patient developed intra-abdominal collection after surgery which resolved with percutaneous drainage. Pathology confirmed a 7 cm ICC, there was evidence of rupture with abscess formation, and adhesion but no direct invasion to duodenum and diaphragm (Fig. 6). The resection margin was clear. No satellite lesion or microvascular invasion. CEA decreased to normal level after surgery. The patient declined adjuvant chemotherapy.

**Figure 7:**
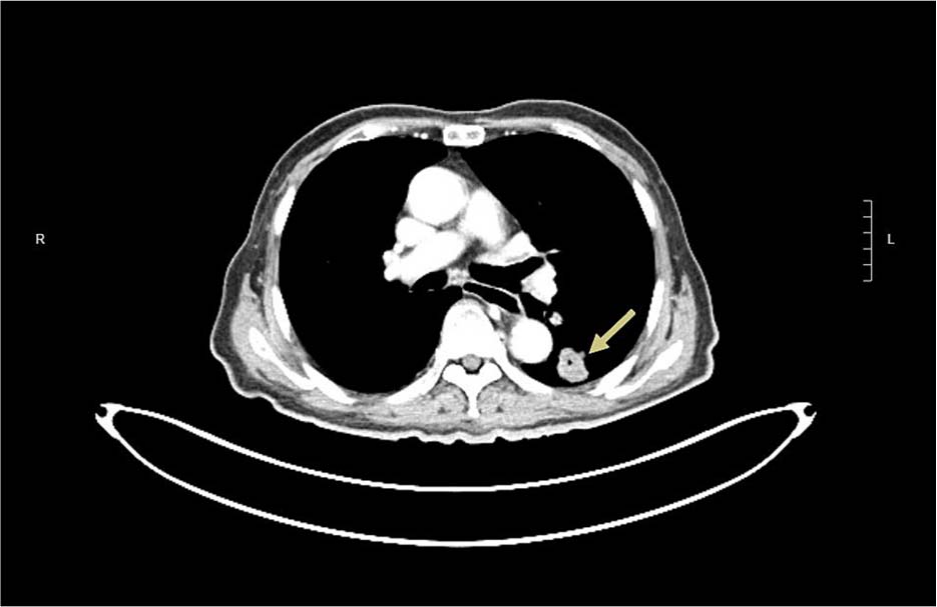
CT revealed a left lower lobe lung lesion (arrow).

**Figure 8:**
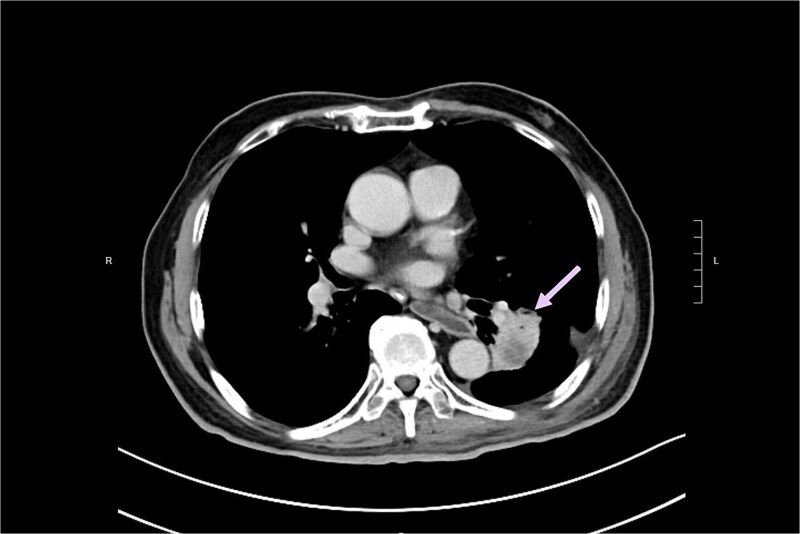
CT revealed post-radiation change in lung lesion (arrow).

The patient was noted to have rising CEA up to 17ug/L at a time over 2 years after operation. PET CT showed a new 2.3 cm hypermetabolic lesion at lower lobe of left lung but no intra-abdominal recurrence (Fig. 7). Biopsy of lung lesion revealed mucinous adenocarcinoma, which could be lung primary or metastatic ICC. As the patient was not a candidate for lung resection, he received stereotactic body radiotherapy (SBRT) for the lung tumor. CEA gradually returned to normal afterwards and repeated CT revealed post-radiation change only at site of tumor (Fig. 8). The patient remained well ˃5 years since initial operation without evidence of further recurrent disease.

## Discussion

Surgery remains the only curative option for ICC though new chemotherapy and immunotherapy for the disease are coming up [2]. Study has shown that provided R0 resection was obtained, extended resection including concomitant visceral or vascular resection could lead to significant survival benefit than those who underwent exploration only [5]. While diaphragmatic resection was commonly done as part of the visceral resection, pericardiectomy was only rarely reported [3, 4]. Primary repair with interrupted sutures of pericardium after pericardiectomy was reported in one case report [6]. We believe that primary closure was only possible with small pericardial defect, otherwise it could lead to pericardial constriction. In our case, we chose to use Gore-Tex for repair as it has been used for long time with good result [7, 8]. It has the advantage of easy availability and lack of reaction between its surface and the epicardium and pericardium. Our patient also did not show any untoward event after repair with Gore-Tex mesh.

There was no actual invasion of ICC to pericardium, diaphragm or duodenum on histological examination in our patient. Actually, it was well known that true infiltration of diaphragm was rare in contrast to the high probability of true infiltration of gastrointestinal structures suspected intraoperatively [3]. One has to balance the added morbidity of en bloc resection of adjacent organ against the chance of R1 resection if actual invasion of adjacent organ really presents. According to previous studies, operative morbidity was not affected by extent of resection [3, 4]. On the other hand, if one abandoned radical resection of ICC in view of suspected involvement of multiple adjacent organs as in this case, this might deny the chance of cure for the patient. The absence of actual invasion to surrounding organ may well explain the ultimate better prognosis in this case.

The initial presentation as upper intestinal bleeding in our patient was also rare. The finding of deep duodenal ulcer and presence of aerobilia on CT were highly suggestive of direct tumor involvement of duodenum by ICC. The subsequent development of a new lung lesion in this case represented a dilemma on nature of lesion. To differentiate the lung lesion as a primary lung cancer or a solitary metastatic ICC was not possible or feasible, hence the practical decision was for SBRT given the patient was a non-surgical candidate, and he responded well to SBRT. He remained recurrence free afterwards and enjoyed normal life. Similar long-term survival after extended surgery including resection of diaphragm, lung and chest wall for ICC has been reported [9].

In summary, our patient demonstrated a long-term survival after initial extended surgery for suspected locally advanced ICC and subsequent radiotherapy for suspected lung recurrence or another lung primary. It shows that a desirable outcome can be achieved with aggressive surgical treatment for locally advanced ICC which is believed to be a deadly disease.
